# Cytoreductive Nephrectomy for Metastatic Renal Cell Carcinoma: A Review of the Historical Literature and Its Role in the Era of Targeted Molecular Therapy

**DOI:** 10.1155/2014/717295

**Published:** 2014-01-23

**Authors:** Muhammad Z. Aslam, P. N. Matthews

**Affiliations:** Department of Urology, University Hospital of Wales, Cardiff CF14 4XW, UK

## Abstract

Renal cell carcinoma presents with metastatic disease in approximately 30% cases. While surgical intervention remains the standard of care for organ confined disease, its role is limited in the management of metastatic disease. Over the last decade, cytoreductive nephrectomy prior to immunotherapy has demonstrated significant improvement in overall survival for appropriately selected patients. This review summarizes the evidence for the role of cytoreductive nephrectomy in combination with immunotherapy and discusses its potential role in the current era of targeted molecular therapy.

## 1. Introduction

Renal cell carcinoma (RCC) accounts for approximately 5% of epithelial cancers worldwide [1] with clear cell RCC representing 85% of these cancers [2]. 30% of patients with RCC are found to have metastatic disease on staging investigations and roughly one-third of patients with organ confined disease undergoing nephrectomy eventually develop metastases [3]. Metastatic RCC (mRCC) is known to have a poor outcome with 2-year median survival rate of only 10–20% [4].

Historically, cytokine based immunotherapies have remained the mainstay of treatment for mRCC [5, 6], until more recently that has been replaced by targeted molecular therapies [7].

Radical nephrectomy as a treatment option in mRCC, (sometimes called debulking or cytoreductive nephrectomy (CRN)) is often indicated as part of an integrated management strategy. It has been previously described in historical series [8], but it was widely accepted as an effective form of treatment in combination with postoperative immunotherapy after the results of 2 prospective randomized trials were published [9, 10]. Previously, nephrectomy had been performed in mRCC patients largely as a palliative measure for control of pain, haemorrhage, paraneoplastic syndromes, and symptoms related to compression of adjacent viscera. It has been reported that nephrectomy performed for these palliative measures can result in spontaneous regression of metastases in up to 4% of cases [11]. Though the exact mechanism of these regression remains unknown, possible explanation could be that nephrectomy might remove a source of tumour-promoting growth factors or immunosuppressive cytokines [12].

## 2. The Historical Series

There was some evidence in historical series that patients treated with immunotherapy respond better if they have previously undergone nephrectomy.

Walther et al. [8] studied 93 patients with the clinical diagnosis of mRCC and manifestations of paraneoplastic syndromes who underwent removal of the primary tumor, as well as debulking of metastases when this could be performed safely. Of the 93 patients, 32% (30) had a second surgical resection in addition to their nephrectomy, in an attempt to deal with the large size of the tumor and invasion of surrounding structures. Postoperative complications were found in 13% of patients while 40% of patients could not receive immunotherapy, because of progression of disease. A preoperative Eastern Oncology Cooperative Group (ECOG) performance status greater than or equal to 2 was the only significant risk factor associated with failure to undergo immunotherapy. The response rate to immunotherapy in the 56 patients receiving interleukin-2 was 27 percent.

Another historical series was based on the UCLA (University of California, Los Angeles) experience on 63 patients. All but one patient had an ECOG performance status of 0 or 1. Postoperative complications were observed in 8 patients (12.7%). Seven patients (11%) could not undergo immunotherapy. Overall, 56 (88%) patients selected underwent immunotherapy. Among these 56 patients, a response rate of 33.9% (7 (12.5%) complete and 12 (21.4%) partial) was observed. Moreover, the 2- and 3-year survival rates were 43% and 38%, respectively [13].

The results of these studies strongly supported the argument for an aggressive approach (surgery combined with IL-2-based immunotherapy) in the management of metastatic RCC.

## 3. The Landmark Studies

The need for multicentre prospective randomized trials with a standardized followup to clarify the role of CRN resulted in the organization of 2 phase III studies supported by South West Oncology Group (SWOG) and European Organization of Research and Treatment of Cancer (EORTC) [9, 10]. Both these studies included patients with synchronous metastatic RCC who were randomized to receive either nephrectomy followed by INF-*α* or INF-*α* monotherapy. The eligibility criteria for both the studies included metastatic RCC with a resectable primary disease, ECOG performance status 0 or 1, no prior radiotherapy or systemic therapy, and adequate end-organ function.

The results of both these trials suggested improved overall survival and time to disease progression in CRN group, though the response rate to immunotherapy did not show any statistically significant difference between the 2 groups. The results of the 2 trials are summarized in Table 1.

## 4. Patient Selection

Patient selection for CRN has remained an area of considerable debate. Though CRN seems to benefit carefully selected patients, it is not a curative procedure and should only be performed in fully consented and informed patients. Many of these patients are elderly and unfit, with very advanced disease and, if subjected to surgery, can experience significant surgical morbidity and mortality, which can delay immunotherapy or prevent the patient from receiving it. This argument was strongly supported by Bennett et al. [14]. Of the 30 patients he studied who underwent CRN, 77% developed disease progression, surgical morbidity, and mortality preventing the administration of IL-2 after CRN. The overall mortality rate was 17% (5 of 30). All of these 5 patients had an ECOG performance status of 1 or higher.

To try and define the ideal patient for CRN, certain inclusion criteria were identified [15]. These included greater than 75% debulking of tumor burden, no central nervous system, bone or liver metastases, adequate pulmonary and cardiac function, ECOG status of 0 or 1, and clear cell histology. Using these criteria on 28 patients, 93% (26 patients) were able to receive systemic therapy with an overall response rate of 39% and median survival of 20.2 months.

Despite this, some patients still do poorly after surgery. In an attempt to improve selection, 7 preoperative factors were described which have negative prognostic effect on outcome and lower survival. These were high serum lactate dehydrogenase, low serum albumin level, symptoms at presentation contributed by metastases, subdiaphragmatic adenopathy, clinical tumor classification >/= T3, liver metastasis, and retroperitoneal adenopathy. Patients who had >/= 4 risk factors did not benefit from CRN [16].

## 5. CRN for Stage IV mRCC

It is known that perioperative mortality affects 10% of patients with stage IV disease [17] as operating with locally advanced disease poses a surgical challenge. In the MD Anderson Cancer centre (MDACC) experience on CRN on 498 patients, 23 have T4NxM1 disease with a median tumour size of 15 cm. The median overall DSS was 6.8 months. The median blood loss was 2.5 litres. 79% patients received postoperative chemotherapy. The median DSS for those who received chemotherapy was 7.1 months versus 2.5 months in those who did not. This study concluded that survival benefit in this subset is unclear and the prognosis in these patients is generally poor [18].

## 6. CRN in Elderly

Kidney cancer is a disease of elderly and the incidence increases linearly with such that patients between 75 and 85 years have the highest incidence, approximately 56/100000 [19]. Elderly group of patients is more susceptible to perioperative complications, due to reduced physiological reserve and an increased incidence of cardiovascular comorbidities. Treating these patients with metastatic disease, thus, is a big challenge. The role of CRN in this group was much clarified by the MDACC experience [20] who studied the outcomes in 24 patients over 75 years and compared them with the younger age group with similar performance status, sex distribution, tumour histology, stage, grade, and size. There were 5 (21%) perioperative deaths in elderly compared to 4 (1.1%) in the younger groups. The blood loss, transfusion rates, and operative time were greater in the patients with mortalities. Interestingly there was no statistically significant difference in the median survival between the 2 groups driving the conclusion that, despite the high risk of morbidity and mortality, CRN could be considered in highly selected and motivated group of elderly with input from experienced surgeons.

## 7. Number of Metastatic Sites

In a retrospective study from UCLA, it was found that the median survival time was 31, 31, and 13 months in the lung, bone, and multiple sites groups, respectively. The response rate to immunotherapy after nephrectomy was 44%, 20%, and 14% in the lung, bone, and multiple organ groups, respectively. Multivariate analysis confirmed that metastatic disease to more than one organ site was associated with poor prognosis [21]. It appears from the results of this study that patients with multiple sites metastases do not have a considerable survival benefit and are best served with nephrectomy only for palliation of symptoms rather than aiming to improve survival.

## 8. CRN in Targeted Molecular Therapy Era

Over the last 4 years, the treatment of mRCC has been revolutionized with the introduction of systemic agents with efficiency much superior to immunotherapy. The majority of clear cell RCCs develop as a result of mutations in the VHL gene [22]. These mutations, through their effects on hypoxia inducible factor (HIF) 1 alpha, lead to the overexpression of multiple hypoxia-responsive proteins that promote angiogenesis and tumour cell growth [23]. This explains the mechanism of action of targeted therapies (TT) using drugs such as sunitinib, sorafenib, temsirolimus, and bevacizumab which all target the angiogenic pathways that are altered by mutations in the VHL genes.

Sunitinib is proven to be the most effective of these new drugs, with both antitumour and antiangiogenic activity. It inhibits multiple tyrosine kinase receptors including VEGF receptor, PDGF receptor, FMS-like tyrosine kinase receptor-3, and c-stem cell factor receptor.

A prospective randomized trial comparing sunitinib versus INF-alpha in patients with mRCC showed superior progression free survival (PFS) in the sunitinib group [7]. An update of this trial showed further prolonged overall survival (OS) in patients treated with sunitinib compared with INF-alpha (median survival of 26.4 versus 21.8 months) [24].

At present, there is no strong evidence to support the role of CRN prior to molecular TT. The evidence provided by nonrandomized studies will always be of poor quality because there will always be a tendency to select fitter patients for the “active” nephrectomy arm.

In a retrospective review based on 314 patients with mRCC, of whom 201 underwent CRN, proved that CRN was associated with median overall survival of 19.8 months compared with 9.4 months without CRN [25]. However the benefit was marginal in patients in the poor prognostic risk group. Another retrospective study from Canada concluded that prior CRN in patients treated with TKIs is associated with improved OS in mRCC on univariate analysis, independent of other prognostic variables [26].

The role of CRN prior to targeted therapy will be clarified by the Clinical Trial to Assess the Importance of Nephrectomy (CARMENA; NCT00930033), which has recently started to recruit a total of 700 patients. Patients with mRCC and good performance status (ECOG PS 0 or 1) who have not had prior systemic therapy or surgical interventions are being randomized to either nephrectomy followed by sunitinib or sunitinib alone [27]. The primary endpoint is OS, with an estimated completion date of 2016.

## 9. Presurgical Targeted Therapy

Presurgical therapy refers to the administration of TT prior to CRN in mRCC. This is to be differentiated from neoadjuvant therapy which refers to the administration of TT in RCC to improve surgical resection of otherwise resectable/nonmetastatic disease. The main goal of presurgical TT is to downsize the primary tumour, improving resectability and decreasing operative risk [28]. It would also allow identification of patients who do not respond to TT and thus may not benefit from surgery. Very limited data is available on the presurgical treatment approach. One of the earliest studies on presurgical treatment assessed efficacy and safety of bevacizumab on 50 patients with mRCC of whom 42 patients underwent nephrectomy. Median PFS was 11.0 months, and OS was 25.4 months. They concluded that presurgical treatment with bevacizumab yields clinical outcomes comparable to postsurgical treatment with antiangiogenic therapy [29].

To assess the timing of surgery in relation to targeted therapy, a prospective randomized EORTC trial has now opened for patients with mRCC to compare the effect of immediate nephrectomy followed by sunitinib versus deferred CRN after 3 courses of presurgical sunitinib (EORTC 30073, SURITIME) [30]. The primary endpoint is PFS; secondary endpoints include OS, morbidity, overall response to treatment in the deferred nephrectomy arm (including the proportion of patients who become unresectable), and the effect of nephrectomy on early progression in both arms.

## 10. Conclusions

Metastatic RCC is a complex disease which carries with it a poor prognosis. The results from 2 randomized trials have clearly demonstrated improved overall survival in patients undergoing nephrectomy prior to systemic therapy, though most of the historical evidence is based on CRN in association with immunotherapy. More recently, management of mRCC has been shifted in favour of several new targeted therapies. This raises the need for prospective randomized trials to outline the efficacy of CRN in combination with targeted therapy. It is hoped that phase III CARMENA and EORTC trials will be able to clarify this issue.

## Figures and Tables

**Table 1:** 

Trial	No. of patients	Median survival (months)		*P* value	Response to therapy (%)		*P *
		IFN	Surgery + IFN		IFN	Surgery + IFN	
SWOG	241	8.1	11.1	0.05	3.3	3.6	NS
EORTC	85	7	17	0.03	12	19	0.38
